# Upadacitinib for treatment of solid facial edema

**DOI:** 10.1016/j.jdcr.2025.11.026

**Published:** 2025-11-24

**Authors:** Rachel K. Greene, Kathryn Hinchee Rodriguez, Milan M. Hirpara, Amanda Duong, Natasha A. Mesinkovska

**Affiliations:** a Department of Dermatology, University of California, Irvine, California; b School of Medicine, California University of Science and Medicine, Colton, California; c School of Medicine, Drexel University College of Medicine, Philadelphia, Pennsylvania

**Keywords:** isotretinoin, JAK inhibitor, Morbihan syndrome, pulse dye laser, solid facial edema, upadacitinib

## Introduction

Morbihan syndrome or solid facial edema (SFE) is a rare complication of both acne and rosacea characterized by cheek and midline facial edema that may alter facial contours. Potential etiologies for this condition are fibrosis secondary to mast cells, as well as impaired lymphatic drainage in the setting of chronic inflammation.^1^^,^^2^ There is currently no standard treatment. SFE responds positively to systemic corticosteroids, isotretinoin, surgical therapy (lymphaticovenous anastomosis), and systemic antibiotics.^2^ The proposed role of perilymphatic granulomas in the pathogenesis of SFE, along with the broader anti-inflammatory properties of Janus kinase (JAK) inhibitors across various cutaneous inflammatory disorders offers new therapeutic insights. Thus, a JAK inhibitor like upadacitinib may be a promising treatment option for SFE in a young woman.

## Case report

**Fig 1:**
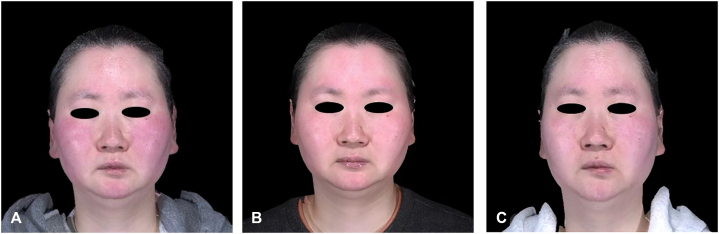
Clinical improvement of a patient with SFE. Baseline while on isotretinoin 20 mg twice daily, botulinum toxin every 4 months, and pulsed dye laser every 2 months **(A)**. At 2 **(B)** and 6 months **(C)** after initiating upadacitinib 30 mg daily, demonstrating progressive reduction in facial erythema and edema.

A 40-year-old Asian female with a history of rosacea presented with a 3-year history of progressive swelling and erythema of face, and ears accompanied by burning and itching. Physical examination revealed erythematous, non-pitting edema of the bilateral centrofacial region and ears with overlying telangiectasias (Fig 1, *A*). She reported that her facial pain was worse at night and often used a battery-operated fan for some relief.

**Fig 2:**
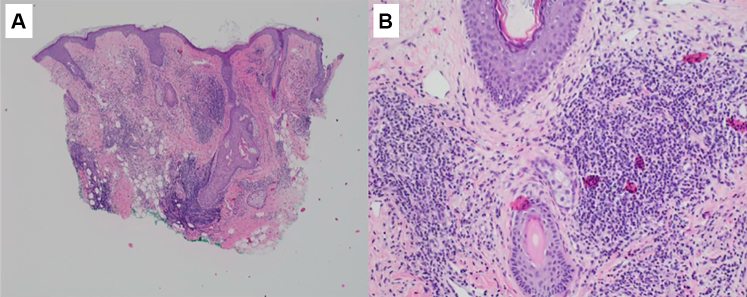
Histopathology of SFE prior to treatment. Low-power view shows a dense superficial and mid-dermal perifollicular inflammatory infiltrate composed of lymphocytes and histiocytes, with prominent dilated blood and lymphatic vessels **(A)**. Higher magnification demonstrates perivascular and perifollicular inflammation with marked dermal edema and vascular dilation **(B)**.

She was treated with topical therapies (pimecrolimus, azelaic acid, salicylic acid, benzoyl peroxide, sulfur soap, adapalene, metronidazole cream, ketamine 10%, amitriptyline 5%, lidocaine 5% cream, hydrocortisone 2.5%, and ketoconazole 2%) and systemic therapies (doxycycline 100 mg, gabapentin 300 mg, and carvedilol 12.5 mg), all of which yielded poor results. A skin biopsy of the left pinna revealed a perifollicular inflammatory infiltrate composed of lymphocytes and histiocytes, as well as widely dilated blood and lymphatic vessels in the upper dermis consistent with SFE (Fig 2). No special stains including colloidal iron or Giemsa were performed.

Autoimmune serology was pursued showing a positive ANA titer (1:160) with a nuclear dense fine speckled pattern. Based on the combination of clinical findings, previous histopathologic results of SFE, the patient was initiated on isotretinoin 20 mg daily. On follow-up, she experienced persistent edema and erythema leading to an isotretinoin dose increase to 20 mg twice daily with minimal improvement.

Over the next 9 months, she had 2 sessions of intradermal botulinum toxin therapy while continuing isotretinoin therapy. This provided temporary clinical relief although symptoms recurred after 8 to 10 week. She received a cumulative isotretinoin dose of 5440 mg (approximately 78.8 mg/kg), along with 3 sessions of pulse dye laser treatment at 595 nm with fluence increasing from 5.5 to 5.75 J/cm^2^. This multimodal treatment approach resulted in slight reduction in erythema and edema. However, her discomfort and heat sensation persisted with an intensity of 9/10, with 10 indicating the most severe symptoms. Therefore, Upadacitinib 30 mg daily was started.

**Fig 3:**
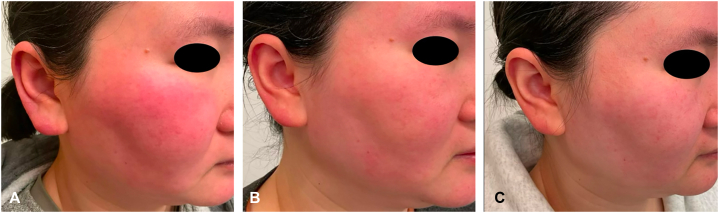
Clinical improvement in a patient with SFE. Baseline before initiating upadacitinib **(A)**. At 2 and 6 months after starting upadacitinib 30 mg daily **(B, C)**, showing marked reduction in facial and ear edema.

After 2 months of Upadacitinib, she reported a decrease in flushing severity to 7/10, with complete resolution of morning episodes. Edema improved to 6/10, and erythema decreased to 6/10. She noted reduced facial and ear edema. While facial burning persisted, she continued to use a fan for symptomatic relief. At 6 months, flushing severity further improved to 4/10, edema to 3/10, and erythema to 2/10. She no longer requires a fan for symptomatic relief (Fig 3). No adverse events were reported from Upadacitinib.

## Discussion

SFE poses a significant therapeutic challenge due to its poorly understood pathophysiology, inconsistent treatment response, and lack of standardized management guidelines. Our case highlights the clinical efficacy of Upadacitinib, isotretinoin and intradermal botulinum toxin which led to improvement in patient-reported outcomes.

Differential diagnoses considered included dermatomyositis, systemic lupus erythematosus, Melkersson–Rosenthal syndrome, thyroid disease, and angioedema. Connective tissue disease was considered given the positive ANA titer (1:320, dense fine speckled pattern); however, the absence of dermal mucin, systemic manifestations, and negative autoantibodies (anti-dsDNA, Sm, RNP, SSA, SSB, Scl-70) argued against lupus or dermatomyositis. Dermatomyositis was further excluded by normal aldolase and creatine kinase levels and lack of Gottron papules or heliotrope rash. Melkersson–Rosenthal syndrome was unlikely due to the absence of granulomatous inflammation, orofacial swelling, or geographic tongue. Thyroid disease can cause periorbital edema, but thyroid-stimulating hormone levels were normal. Angioedema was ruled out clinically with no acute swelling, urticaria, or complement abnormalities. Collectively, findings supported a non-granulomatous inflammatory process consistent with SFE.

The exact etiology of SFE remains unknown. One potential etiology is chronic inflammatory processes may cause fibrosis and Upadacitinib, a JAK1 inhibitor downregulates the inflammatory mediators implicated in both rosacea and chronic dermal edema (IL-4, IL-6, IL-13, IL-22, and IL-31) which can help with resolution of obstruction of the deep dermal lymphatic vessels resulting in fluid accumulation.^3^ Compared to other JAK inhibitors, upadacitinib offers a more targeted suppression of type II cytokine signaling. This may account for the relatively rapid clinical onset in this case within 2 months, versus prior reports using abrocitinib.^3^^,^^4^

SFE is considered a complication of rosacea and shares a similar pathophysiology pathway thereby, the observed effectiveness of Upadacitinib to treat SFE appears consistent. In our case, the patient experienced symptomatic relief after 2 months of upadacitinib treatment. Previous cases report described clinical relief in SFE with other JAK inhibitors such as Abrocitinib 200 mg daily after 7.5 months.^5^ While some symptoms may persist, the overall improvement in this patients’ quality of life underscores the therapeutic potential for JAK inhibitors in managing SFE. Yet, the difference shows potential variability in therapeutic response across the JAK inhibitors class and highlights the need to select the most effective agent.

In addition to upadacitinib, our regimen included intradermal botulinum toxin, which likely contributed to improvements in erythema and flushing through a neuromodulatory pathway. Trigeminal nerve activation and neuropeptide release (eg, substance P, CGRP) can trigger vasodilation and mast cell degranulation, perpetuating inflammation.^2^ As a neuromodulator, botulinum toxin acts at the neuromuscular junction to inhibit the release of these neuropeptides, thereby reducing neurogenic inflammation, suppressing mast cell activation, and mitigating the associated clinical response.^2^ The inclusion of botulinum toxin in our regimen may have contributed to some observed improvement in erythema and morning flushing.

Upadacitinib provided significant symptomatic improvement along with a favorable side effect profile. Larger and long-term studies are needed in the future to confirm our findings.

## Conflicts of interest

None disclosed.

## References

[1] Heibel H.D., Heibel M.D., Cockerell C.J. (2020). Successful treatment of solid persistent facial edema with isotretinoin and compression therapy. JAAD Case Rep.

[2] van der Linden M.M., Arents B.W., van Zuuren E.J. (2023). Diagnosis and treatment of Morbihan's disease: a practical approach based on review of the literature. J Clin Aesthet Dermatol.

[3] Wilkin J.K. (1994). Rosacea. pathophysiology and treatment. Arch Dermatol.

[4] Hopkinson D., Moradi Tuchayi S., Alinia H., Feldman S.R. (2015). Assessment of rosacea severity: a review of evaluation methods used in clinical trials. J Am Acad Dermatol.

[5] De Greef A., Baeck M. (2025). Abrocitinib for treatment of solid facial edema. JAAD Case Rep.

